# Psychosocial health of school-aged children during the initial COVID-19 safer-at-home school mandates in Florida: a cross-sectional study

**DOI:** 10.1186/s12889-021-10540-2

**Published:** 2021-03-29

**Authors:** Sarah L. McKune, Daniel Acosta, Nick Diaz, Kaitlin Brittain, Diana Joyce- Beaulieu, Anthony T. Maurelli, Eric J. Nelson

**Affiliations:** 1 Departments of Environmental and Global Health, College of Public Health and Health Professions, University of Florida, Gainesville, FL USA; 2 Department of Special Education, School Psychology, & Early Childhood Studies, College of Education, University of Florida, Gainesville, FL USA; 3 Emerging Pathogens Institute, University of Florida, Gainesville, FL USA; 4 Department of Pediatrics, College of Medicine, University of Florida, Gainesville, FL USA

**Keywords:** COVID-19, Mental health, Psychosocial impacts, Vulnerable population, Pandemic

## Abstract

**Background:**

Given the emerging literature regarding the impacts of lockdown measures on mental health, this study aims to describe the psychosocial health of school-aged children and adolescents during the COVID-19 Safer-at-Home School mandates.

**Methods:**

A cross-sectional study was conducted in April 2020 (*n* = 280) among K-12 students at a research school in North Central Florida. Bivariate analysis and logistic and multinomial logistic regression models were used to examine socio-demographic and knowledge, attitude, and practice (KAP) predictors of indicators of anxiety-related, depressive, and obsessive-compulsive disorder(OCD)-related symptoms. Outcomes (anxiety, OCD, and depressive related symptoms) were measured by indices generated based on reported symptoms associated with each psychosocial outcome.

**Results:**

Loss of household income was associated with increased risk for all three index-based outcomes: depressive symptoms [aOR = 3.130, 95% CI = (1.41–6.97)], anxiety-related symptoms [aOR = 2.531, 95%CI = (1.154–5.551)], and OCD-related symptoms [aOR = 2.90, 95%CI = (1.32–6.36)]. Being female was associated with being at higher risk for depressive symptoms [aOR = 1.72, 95% CI = (1.02–2.93)], anxiety-related symptoms [aOR = 1.75, 95% CI = (1.04–2.97)], and OCD-related symptoms [aOR = 1.764, 95%CI = (1.027–3.028)]. Parental practices protective against COVID-19 were associated with children being at higher risk of depressive symptoms [aOR = 1.55, 95% CI = (1.04–2.31)]. Lower school level was associated with children being at higher risk of anxiety-related and OCD-related symptoms.

**Conclusions:**

As the COVID-19 pandemic continues, schools should prioritize mental health interventions that target younger, female students, and children of families with income loss. Limiting the spread of COVID-19 through school closure may exacerbate negative psychosocial health outcomes in children, thus school administrators should move quickly to target those at greatest risk.

**Supplementary Information:**

The online version contains supplementary material available at 10.1186/s12889-021-10540-2.

## Background

Social distancing is one primary public health intervention to reduce the spread of SARS-CoV-2, the virus that causes COVID-19. In the Spring of 2020, school closures, limits on the number of individuals allowed at social gatherings, and closing restaurants, bars, and businesses rapidly reduced transmission and flattened the epidemiologic curve in many parts of the US, including Florida. Though children appear to be at less risk of severe illness and mortality associated with SARS-CoV-2 infection [1], these interventions significantly disrupted the lives of children and families – and not equally. Disparities in health care, education, and wealth, which are prevalent across the United States, put minorities and disadvantaged groups at higher risk for myriad negative outcomes associated with the pandemic.

Scientists urged the research community early in the pandemic to prioritize high quality data on mental health and psychosocial effects of the COVID-19 pandemic [2, 3]. Past evidence has shown significant psychological effects on children during disasters [4], and reports of psychosocial distress in children and adolescents have increased in the COVID-19 pandemic compared to pre-pandemic baseline [5, 6]. COVID-19 related hardships will affect psychological wellbeing of children, with greater impact on those who have pre-existing mental illness or who live in households facing larger economic distress [6, 7]. Findings from early studies in China found an increase in the prevalence of children reporting symptoms of depression and anxiety during home confinement [8]. A similar study of the general population in China found that some preventive practices were associated with reporting symptoms of poor mental health [9]. Preventive practices are the goal of public health interventions that leverage the knowledge, attitude, and practice (KAP) pathway to improve various health outcomes [10–12]. Misinformation was a public health concern at the beginning of the COVID-19 pandemic [13], but how knowledge was affecting safe practices or psychosocial health is still unclear. Based on literature examining KAP and psychological distress during other crises/pandemics in the general population, a study from the H7N9 pandemic in Hong Kong showed that attitudes valuing improved personal hygiene were associated with higher anxiety levels [14]. A more recent study found association between knowledge about certain COVID-19 related items, such as transmission, and depression, stress, and anxiety [15].

COVID-19 psychosocial data remain limited, especially data representative of racial and ethnically diverse populations of children in the US. Given that minority communities and communities of color in the US have experienced much higher rates of infection [16], the distribution of any psychological toll associated with COVID-19, may be disproportionately experienced by children of these communities, thus exacerbating inequalities in the negative health impacts of COVID-19.

Outside of the COVID-19 pandemic, there exists a robust literature on age, sex, race, and ethnic disparities in pediatric mental health, including differences in underlying risk factors associated with mental health problems, need for mental health services, appropriateness of diagnostic tools, and access to and uptake of mental health services and interventions [17–23]. Underlying factors such as socioeconomic status and loss of income are associated with mental health diagnoses. Importantly, research in this space has also uncovered a *race paradox* in mental health, where Blacks, who suffer greater negative exposure to stress, discrimination, and poverty, often have mental health outcomes that are better than or comparable to their white counterparts - an area of ongoing research [24–27]. Like any sub-population, children are not a monolith, and research is needed to understand how these social stratifications may differently affect them. This information is necessary to tailor and target appropriate interventions that may prevent or mitigate the varied negative psychosocial impacts of the COVID-19 pandemic.

The aim of this study is to describe the psychosocial health of a population of school-aged children during the early phase of the COVID-19 pandemic, when schools were closed and children were rapidly transitioned to online learning. The study examines social groups (sex, age, race, and ethnicity) and COVID-19 related parental KAP as risk factors for psychosocial health outcomes, including anxiety-related, depressive, and obsessive-compulsive disorder (OCD)-related symptoms among school-aged (K-12) children at the beginning of the pandemic. This information will assist school administrators, public health practitioners, and policy makers in designing evidence-based, targeted interventions to address the psychosocial needs of the children during the ongoing COVID-19 pandemic.

## Methods

### Study design

This study was part of a broad multi-disciplinary study to identify determinants of SARS-CoV-2 infection and transmission among diverse populations in Florida starting in March 2020. In order to better understand COVID-19 in children, we established a prospective cohort study in collaboration with a K-12 public school with initial collection points at baseline, 6, and 12 months. At each phase of the school-based study, researchers included/will include online survey-based questions that reflect the school’s desire to understand how children and their families are being affected at each phase of the COVID-19 pandemic (See Additional file 1). The survey is coupled with the COVID-19 research tests, which include collection of oropharyngeal swabs (PCR for SARS-CoV-2 detection) and finger sticks (ELISA for antibodies) from student participants at a drive-thru testing site. While laboratory data associated with the cohort study will be published independently, we present here results from a cross-sectional analysis of socio-demographics, parental COVID-19 related KAP, and indicators of psychosocial health outcomes in children collected at the baseline timepoint of April 2020.

At the K-12 school, data collection captured the psychosocial effects of COVID-19 on school-aged children and their parents. This school is mandated to have a student body that reflects the demographic composition of the school-aged population of the State of Florida, as indicated by gender, race/ethnicity, and family income. Parents/guardians of all current students (*N* = 1178) were invited by email to have their child participate in the study. No compensation was offered to participants. However, participation included the COVID-19 testing, which had been very well-received within other sub-populations of the parent study, likely based on the limited availability of testing at the time. Recruitment materials included reminder emails and a website with additional information about the study. Those who were interested were able to consent/assent to participation online via a HIPAA compliant interface connected to REDCap (Vanderbilt University). Parental consent was required for all children under the age of 18; and all children over the age of eight assented for themselves online. All data were deidentified prior to analysis and were stored on secured servers to ensure the protection of participants (REDCap). The study was approved by the University of Florida Institutional Review Board, protocol IRB202001345.

### Variables and statistical analyses

The demographics section of the survey included questions about child’s age, sex, race, ethnicity, parental occupation (whether a parent was medical/frontline worker or not), and the loss of household income due to COVID-19 (self-reported binary variable on whether the household had lost income due to COVID-19). The parental COVID-19 related KAP questions, originally included in the survey as potential risk factors for infection, are included in this cross sectional analysis to explore potential association between parental COVID-19 related KAP and child psychosocial outcomes. The KAP section consisted of 14 knowledge, eight attitude, and five practice questions. A knowledge score was created by assigning one (1) to each correctly answered knowledge question about COVID-19 (transmission, prevention, and/or general information) and summing them for a total possible score of 14. Similarly, attitude and practice scores were categorized as protective and unprotective, with protective attitudes and practices coded as one (1) and all others as zero (0), and summed, for respective possible scores of 8 and 5. Questions for the KAP section were based on existing literature of KAP and COVID-19, which at the time was limited to a few published studies [28, 29].

The questions used to assess psychosocial outcomes evaluated the self-reported frequency of symptoms associated with three internalizing disorders: depression, anxiety, and OCD (Table 1). These emotional and behavioral disorders were included based on their critical importance to school aged children during crises and disasters [5, 30, 31] and the partnering school’s interest in these data to target and develop appropriate interventions. Symptoms of psychosocial health were collected rather than diagnosed measures, as the original baseline study was designed not to study psychosocial outcomes but infection in children and families. In an effort to ensure these questions were included in the in the survey, which was quite long due to inclusion of a number of research modules, an abbreviated list of symptoms commonly associated with these diagnoses was used. Research outcomes reported in this paper (anxiety-related, OCD-related, and depressive symptoms) are indices based on reported symptoms. Each symptom was evaluated using a set of categorical questions (5-point Likert scale). All questions used age-appropriate language and response options (e.g. 1-Never, 2-A Little, 3-Sometimes, 4-A Lot, or 5-Always/Constantly). Within each group of questions, if a participant’s responses were all 1 s or 2 s, the child was categorized *Not at Risk*; everyone else was considered *At Risk* and further categorized as *High*, *Medium*, or *Low Risk*, using the frequency of their highest response options (see Likert scale above). Participants who answered “a lot” (4) to two or more questions in a group or “always/constantly” (5) to any one of the questions in the group were considered *High Risk*; participants whose highest Likert scale response was (4), indicating “a lot” only once, with all other questions at a 3 or below, were considered *Medium Risk*; and any participant whose highest response was (3), “sometimes” was considered *Low Risk*. Each group of questions used to assess the risk of these psychosocial health outcomes and the methodology described here to develop ordinal variables associated with each outcome was designed by a team of school psychologists with the aim of identifying groups of students at risk of developing one of the specific psychosocial outcomes mentioned above. The six primary outcome variables in this study are *At Risk* (using *No Risk as reference (REF)*) and *High Risk* (REF *No Risk*) of anxiety-related, depressive, and OCD-related symptoms. A summary outcome variable, *Any Risk* indicates if a child presents as *At Risk* for any of the three psychosocial outcomes assessed here.

**Table 1:** Reliability measures for age specific psychosocial indicators

Indicator	Questions	Cronbachs alpha
Depression symptoms for children over 13	I feel hopeless and sad (about the virus)	0.634
	I have trouble eating or sleeping	
	I find myself crying a lot	
	I have a stomachache/headache	
	It’s hard for me to think a long time	
Anxiety symptoms for children over 13	I have trouble eating or sleeping	0.723
	I feel worried or nervous (about the virus)	
	It is hard to stop my thoughts (about the virus)	
	I cannot stop worrying (about the virus)	
	It’s hard for me to think a long time	
OCD symptoms for children over 13	I feel worried or nervous (about the virus)	0.785
	It is hard to stop my thoughts (about the virus)	
	I cannot stop worrying (about the virus)	
	I am very scared of getting dirty	
	I have to wash my hands, over and over to feel better	
Depression Over 13	Sadness, feeling down, low mood, feeling fatigued	0.83
	Feelings of hopelessness, worthlessness, emptiness, or not being a good person	
	Decreased pleasure from things that used to be fun, feeling that life is not much fun	
	Being easily annoyed or irritable, feelings of dread like something awful might happen	
Anxiety Over 13	Feeling worried, nervous, panicky, tense, keyed-up	0.831
	Not being able to stop worrying or controlling your worry	
	Being easily annoyed or irritable, feelings of dread like something awful might happen	
	Felt a racing heart, shaky sweaty, or had trouble breathing	
OCD Over 13	Not being able to stop worrying or controlling your worry	0.725
	Constant thoughts about avoiding germs	
	Fixation with washing your hands throughout the day	
	Sudden moments of fear or terror because you couldn’t get rid of the germs	

Bivariate analysis was conducted to test for association with parental knowledge, attitude, and practice scores, demographic variables, and parental occupation. An additional bivariate analysis of each individual KAP question was used to identify association between any KAP item (question) and race/ethnicity using Fisher’s Exact Test and logistic regression. Further analysis was conducted using logistic and multinomial logistic regression models to examine predictors of At Risk (REF No Risk) and High Risk (REF No Risk), respectively, for each depressive, anxiety-related, and OCD-related symptoms. The covariates included in the models were race/ethnicity, sex, school level, household loss of income during the pandemic, parental occupation, knowledge score, attitude score, and practice score. These covariates were selected in order to identify individual, parental, and household level characteristics of students who were in greatest need of psychosocial support during the pandemic. Of 390 surveys initiated online, 17 were duplicate surveys (parents initiated twice for the same child, with only one complete) and 93 were initiated but not completed; thus 280 K-12 students completed surveys. Only complete surveys were included in the analysis. Because the survey was conducted online with fields required to advance and survey completion required to participate in the testing arm of the study, there were no missing values in the surveys completed online. Therefore 280 children are included in the analysis.

Survey data were collected in REDCap and analyzed in R Software (version 4.0.0) and SPSS (version 26).

## Results

A total of 280 students were enrolled out of student body of 1178 (23.7%). Table 2 presents the demographic characteristic of the sample and the prevalence of symptoms of the three psychosocial health outcomes considered in this study (depressive, anxiety-related, or OCD-related symptoms). The prevalence of individuals presenting with symptoms associated with being at *Any Risk* of anxiety, depression, or OCD in the sample was 47.1%. For anxiety-related symptoms, 34.6% of students were identified as being *At Risk* and 8.6% at *High Risk.* For depressive symptoms, 35.4% were *At Risk* and 6.8% at *High Risk.* For OCD-related symptoms, 32.1% were *At Risk* and 8.9% at *High Risk.* The study population was nearly two-thirds White (non-Hispanic), a fifth Hispanic (regardless of race), and 9% Black (non-Hispanic). Participants were 48% male and distributed across high school (40%), middle school (29%) and primary school (31%). The prevalence of those presenting with symptoms meeting the definition of both *Any Risk* and *High Risk* of depression, anxiety, and OCD, were consistently greater among females and primary school students.

**Table 2:** Demographic characteristics of the study population (ordered by representation level on the sample) (n = 280)

	Study Sample	Anxiety-related symptoms % (n)		Depressive symptoms % (n)		OCD-related symptoms % (n)	
	% (n)	At Risk	High Risk	At Risk	High Risk	At Risk	High Risk
**Prevalence of risk (% from the sample)**	47.1% (132)	34.6% (97)	8.6% (24)	35.4% (99)	6.8% (19)	32.1% (90)	8.9% (25)
**Race/Ethnicity**							
White	62% (174)	36.8% (64)	8.6% (15)	37.9% (66)	5.2% (9)	31.0% (54)	8.0% (14)
Hispanic	19% (53)	26.4% (14)	9.4% (5)	28.3% (15)	11.3% (6)	32.1% (17)	7.5% (4)
Multiracial	10% (29)	37.9% (11)	10.3% (3)	31.0% (9)	10.3% (3)	37.9% (11)	10.3% (3)
Black	9% (24)	33.3% (8)	4.2% (1)	37.5% (9)	4.2% (1)	33.3% (8)	16.7% (4)
**Sex**							
Female	51.8% (145)	40.0% (58)*	10.3% (15)	40.0% (58)	9.7% (14)	37.9% (55)*	11.7% (17)
Male	48% (135)	28.9% (39)*	6.7% (9)	30.4% (41)	3.7% (5)	25.9% (35)*	5.9% (8)
**School Level**							
High School	39.6% (111)	27.0% (30)	6.3% (7)	30.6% (34)	8.1% (9)	27.9% (31)	6.3% (7)
Middle School	29.3%(82)	36.6% (30)	6.1% (5)	37.8% (31)	4.9% (4)	28.0% (23)	7.3% (6)
Primary School	31.1% (87)	42.5% (37)	13.8% (12)	39.1% (34)	6.9% (6)	41.4% (36)	13.8% (12)
**Household loss of income due to COVID-19**							
No	88.2% (247)	32.4% (80)*	8.9% (22)	32.8% (81)*	6.1% (15)	29.6% (73)*	8.1% (20)
Yes	11.8% (33)	51.5% (17)*	6.1% (2)	54.5% (18)*	12.1% (4)	51.5% (17)*	15.2% (5)
**Parent working on frontline**							
No	64.6% (181)	38.1% (69)	10.5% (19)	40.3% (73)*	7.7% (14)	35.9% (65)	11.0% (20)
Yes	35.4% (99)	28.3% (28)	5.1% (5)	26.3% (26)*	5.1% (5)	25.3% (25)	5.1%(5)
**KAP Scores (average)**							
Knowledge Score	12.79	12.81	12.83	12.76	13.00	12.81	12.44
Attitude Score	6.87	6.87	7.33	6.83	7.26	7.00	7.08
Practice Score	4.35	4.37	4.5	4.45	4.42	4.40	4.36

*p-value < 0.05

Parental knowledge about COVID-19 was high, with 31.4% answering all questions correctly, and only 13.6% answering three or more questions incorrectly (Table 3). Attitudes had similar results, with most parents expressing agreement with protective attitudes. Preventative practices were also high, with at least 90% of respondents reporting increased hand washing, avoiding physical contact with those outside their home, and adhering to social distancing guidelines (Table 4).

**Table 3:** Knowledge Results. *P*-values are for Fisher’s Exact Test comparing knowledge answers and race/ethnicity

Knowledge Results			
Questions		*n* = 280	
	Correct	Incorrect	*p*-value
	No (%)	No (%)	
The main clinical symptoms of COVID-19 are fever, fatigue, dry cough, and muscle aches.	265 (94.6%)	15 (5.4%)	0.589
Unlike the common cold, stuffy nose, runny nose, and sneezing are less common in persons infected with the COVID-19 virus.	220 (78.6%)	60 (21.4%)	0.747
There currently is no effective cure for COVID-19, but early symptomatic and supportive treatment can help most patients recover from the infection.	265 (94.6%)	15 (5.4%)	0.016*
Antibiotics can be used to treat COVID-19^a^	210 (75.0%)	70 (25%)	0.732
Not all persons with COVID-19 will develop to severe cases. Those who are elderly and have chronic illnesses are more likely to be severe cases.	277 (98.9%)	3 (1.1%)	1.000
People of all racial and ethnic groups can become infected with the COVID-19 virus.	276 (98.6%)	4 (1.4%)	0.818
Most people who are infected with the COVID-19 virus recover from it	258 (92.1%)	22 (7.9%)	0.609
Handwashing can help reduce transmission of the COVID-19 virus.	279 (99.6%)	1 (0.4%)	1.000
Persons with COVID-19 cannot pass the virus to others if they do not have symptoms.	261 (93.2%)	19 (6.8%)	0.823
The COVID-19 virus spreads via respiratory droplets of infected individuals	269 (96.1%)	11 (3.9%)	0.657
Ordinary residents can wear general medical masks to prevent infection by the COVID-19 virus.	173 (61.8%)	107 (38.2%)	0.015*
It is not necessary for children and young adults to take measures to prevent infection by the COVID-19 virus.	272 (97.1%)	8 (2.9%)	0.533
Isolation and treatment of people who are infected with the COVID-19 virus are effective ways to reduce the spread of the virus.	278 (99.3%)	2 (0.7%)	1.000
People who have contact with someone infected with the COVID-19 virus should be immediately isolated in a proper place. In general, the observation period is 14 days.	278 (99.3%)	2 (0.7%)	0.379
**Cumulative Knowledge Score**		n = 280	
**Scores out of 14**		No.	%
**< 11**		8	2.9%
**11**		30	10.7%
**12**		55	19.6%
**13**		99	35.4%

*p-value< 0.05

^a^This item is false. The survey was developed and implemented prior to the use of hydroxychloroquine, azithromycin, or remdesivir in experimental trials

**Table 4:** Comparison of protective and un-protective attitudes and practices with race/ethnicity

Attitudes	*n* = 280			
	Agree	Disagree	Don’t know	*p*-value
	No (%)	No (%)	No (%)	
I am worried about getting infected with the COVID-19 virus.	176 (63%)	82 (29.3%)	22 (7.9%)	0.030^a^
I feel confident I can prevent myself and my family from becoming infected with the COVID-19 virus if it becomes more widespread in Florida.	151 (53.9%)	64 (22.9%)	65 (23.2%)	0.275
I know what actions to take to prevent myself and my family from becoming infected with the COVID-19 virus.	256 (92.5%)	9 (3.2%)	12 (4.3%)	1.000
I support a government-imposed mandatory quarantine for those who are infected with the COVID-19 virus.	266 (95.0%)	4 (1.4%)	10 (3.6%)	0.697
I support voluntary home quarantine for up to 2 weeks for people who have been in contact with someone who has COVID-19.	275 (98.2%)	1 (0.4%)	4 (1.8%)	0.064
I support postponing or canceling mass gatherings such as concerts, festivals, and sporting events.	268 (95.7%)	4 (1.4%)	8 (2.9%)	0.325
I support closure of K-12 schools if any student, staff member, or teacher is found to have COVID-19	251 (89.6%)	11 (3.9%)	18 (6.4%)	0.184
If I were exposed to and could possibly be infected with the COVID-19 virus, I would be willing to quarantine myself at home for 2 weeks until I was sure I was not infected in order to prevent others from getting COVID-19 from me.	278 (99.3%)	0 (0%)	2 (0.7%)	0.103
Practice		Yes	No	*p*-value
		No (%)	No (%)	
In recent days, are you washing your hands with soap and water more often than normal?		262 (93.6%)	18 (6.4%)	1.000
In recent days, are you using more disinfectants, such as hand sanitizers and cloth wipes?		252 (90.0%)	28 (10.0%)	0.878
In recent days, are you avoiding shaking hands or other physical contact with others outside your home?		277 (98.9%)	3 (1.1%)	1.000
In recent days, have you adhered to other social distancing guidelines, such as avoiding meetings of more than 10 people and keeping a distance of 6 ft apart?		279 (99.6%)	1 (0.4%)	1.000
In recent days, have you bought larger amounts of staple foods (flour, sugar, pasta, rice, canned food) than normal?		148 (52.9%)	132 (47.1%)	0.042^a^

^a^Fisher’s Exact Test. *p*-value< 0.05

Though there were no significant differences in parental knowledge, attitude, or practice scores by race and ethnicity, there were significant race/ethnic differences for specific items (questions) within those scores. These included 1) knowledge about mask usage, 2) knowledge about COVID-19 treatment and cure, 3) feeling worried about getting infected by the virus, and 4) stockpiling staple foods (Tables 3 and 4). Non-Hispanic White respondents were less worried overall about getting infected with the virus compared to Non-Hispanic Blacks (*p*-value = 0.040) or Hispanics (*p*-value = 0.026). Non-Hispanic White respondents were more likely to answer incorrectly the question about mask usage than Non-Hispanic Blacks (*p*-value = 0.014) or Hispanics (*p*-value = 0.034). There were no significant differences between Multiracial/Other and Non-Hispanic White respondents in knowledge about mask usage (*p*-value = 0.30) or being worried about infection with the virus (*p*-value = 0.21). Among those practices assessed, no significant difference by race/ethnicity was found for social distancing, handwashing, or general hygiene; however, Hispanic respondents were less likely to report purchasing larger amounts of staple foods than normal when compared to Non-Hispanic Whites (*p*-value = 0.08).

Loss of household income was significantly associated with students being *At Risk* of depressive symptoms [aOR = 3.130, 95% CI = (1.41–6.97)], anxiety-related symptoms [aOR = 2.531, 95%CI = (1.154–5.551)], and OCD-related symptoms [aOR = 2.90, 95%CI = (1.32–6.36)], see Table 5. Being female was significantly associated with being *At Risk* for depressive symptoms [aOR = 1.72, 95% CI = (1.02–2.93)], anxiety-related symptoms [aOR = 1.75, 95% CI = (1.04–2.97)], and OCD-related symptoms [aOR = 1.764, 95%CI = (1.027–3.028)]. School level was significantly associated with being *At Risk* and *High Risk* of both anxiety-related and OCD-related symptoms (Table 5). Those in primary school and middle school are more likely to be *At Risk* for anxiety-related and OCD-related symptoms than students in high school, and those in primary school being more likely to be at *High Risk* for anxiety-related and OCD-related symptoms than those in high school. A family’s COVID-19 Practice score was significantly associated with a child being *At Risk* for depressive symptoms [aOR = 1.55, 95% CI = (1.04–2.31), Table 5]; those families who followed stricter protective practices were more likely to have a child who presented as *At Risk* for depressive symptoms.

**Table 5:** Children At Risk and at High Risk for depressive, anxiety-related, and OCD-related symptoms

	Anxiety-related symptoms		Depressive symptoms		OCD-related symptoms	
	At Risk	High Risk	At Risk	High Risk	At Risk	High Risk
**Prevalence of risk (% from the sample)**	34.6%	8.6%	35.4%	6.8%	32.1%	8.9%
**Covariates**	aOR At Risk (95% C.I.)^a^	aOR High Risk (95% C.I.)^b^	aOR At Risk (95% C.I.)^a^	aOR High Risk (95% C.I.)^b^	aOR At Risk (95% C.I.)^a^	aOR High Risk (95% C.I.)^b^
**Race/Ethnicity**						
White	Ref (NA)	Ref (NA)	Ref (NA)	Ref (NA)	Ref (NA)	Ref (NA)
Black	0.758 (0.29–1.97)	0.34 (0.04–2.96)	0.97 (0.39–2.48)	0.74 (0.082–6.78)	1.01 (0.39–2.66)	2.17 (0.57–8.37)
Hispanic	0.589 (0.28–1.22)	0.82 (0.26–2.64)	0.68 (0.33–1.42)	1.83 (0.56–5.99)	1.02 (0.49–2.08)	0.79 (0.22–2.82)
Multiracial	1.034 (0.43–2.49)	1.47 (0.34–6.36)	0.78 (0.31–1.96)	2.53 (0.49–11.41)	1.41 (0.58–3.43)	1.55 (0.35–6.87)
**Sex**						
Male	Ref (NA)	Ref (NA)	Ref (NA)	Ref (NA)	Ref (NA)	Ref (NA)
Female	1.753 (1.04–2.97)*	1.93 (0.76–4.88)	1.72 (1.02–2.93)*	2.93 (0.970–8.84)	1.76 (1.03–3.03)*	2.58 (0.99–6.68)
**School Level**						
High School	Ref (NA)	Ref (NA)	Ref (NA)	Ref (NA)	Ref (NA)	Ref (NA)
Middle School	1.82 (0.95–3.56)	1.26 (0.37–4.33)	1.67 (0.88–3.18)	0.83 (0.23–2.99)	1.16 (0.59–2.26)	1.46 (0.44–4.87)
Primary School	2.32 (1.23–4.36)*	2.94 (1.04–8.30)*	1.63 (0.88–3.07)	1.03 (0.33–3.26)	2.01 (1.07–3.77)*	3.10 (1.05–9.12)*
**Household loss of income due to COVID-19**						
No	Ref (NA)	Ref (NA)	Ref (NA)	Ref (NA)	Ref (NA)	Ref (NA)
Yes	2.53 (1.15–5.55) *	1.17 (0.23–5.8)	3.13 (1.41–6.97) *	3.74 (1.00–14.01) *	2.90 (1.32–6.36) *	3.19 (0.91–11.12)
**Parent working on frontline**						
No	Ref (NA)	NA	Ref (NA)	Ref (NA)	Ref	NA
Yes	0.66 (0.37–1.18)	0.41 (0.13–1.25)	0.59 (0.33–1.07)	0.43 (0.13–1.45)	0.59 (0.32–1.09)	0.42 (0.14–1.31)
**KAP Scores**						
Knowledge Score	1.05 (0.83–1.32)	0.96 (0.64–1.43)	0.96 (0.77–1.21)	1.11 (0.69–1.76)	0.89 (0.77–1.25)	0.72 (0.50–1.04)
Attitude Score	0.97 (0.75–1.25)	1.82 (0.97–3.43)	0.90 (0.71–1.16)	1.51 (0.79–2.89)	0.25 (0.89–1.56)	1.46 (0.87–2.44)
Practice Score	1.08 (0.75–1.58)	1.19 (0.57–2.52)	1.55 (1.04–2.31)*	1.34 (0.63–2.86)	0.42 (0.79–1.74)	1.n (0.57–2.22)

^a^Binary Logistic Regression was used to model At Risk, using No Risk as the reference category

^b^Multinomial Logistic Regression was used to model High Risk using No Risk as the reference category

^*^ *p*-value< 0.05

## Discussion

This study aimed to identify risk factors associated with school-aged children presenting symptoms of anxiety, OCD, and depression during the early days of the COVID-19 pandemic. Almost half of the students that participated in the study showed symptoms consistent with being at risk for anxiety, OCD, or depression. Results indicate that students in households who had experienced COVID-19 related loss of income by April 2020, students who were female, and students in primary school were among those at greatest risk. In addition, families who reported more COVID-19 protective practices were also more likely to have children who present at risk for depressive symptoms.

Loss of household income was associated with mental distress across the sample population, which is consistent with other studies, suggesting that the economic impacts of the lockdown are an important trigger for mental distress in children [3, 7, 9].. Economic hardship alone might be associated with increased risk of socioemotional problems in children, exacerbated by their parents’ response to the situation [18]. Sex was an important risk factor, as females were more likely to be at risk for depressive, anxiety-related, and OCD-related symptoms. This finding echoes results from post-disaster related studies, where being female was associated with increased psychosocial risk [32]. This could also be related to reporting, as females are more likely than males to report their risk and/or clinical symptoms of depression [33–35]. Results also suggest that children in primary school are more likely to present as at high risk for anxiety-related and OCD-related symptoms, making them priority groups for interventions at schools. Though this may seemingly contradict evidence that psychological disorders increase in adolescence [36, 37], it is in keeping with studies on the psychosocial impact of disasters and crises in children, which indicate that this population is susceptible to experiencing distress of this type during crises [30, 31]. Overall, the results of the study indicate that COVID-19’s psychosocial effects on school-aged children are in keeping with crises and disasters.

Parental reporting of practices protective against SARS-CoV-2 infection and transmission was associated with children presenting as At Risk for depressive symptoms. Distancing and isolation can increase stress, which can aggravate feelings of loneliness and impact long term health [3]. This is consistent with studies in China and Turkey that found increased depressive symptoms among children due to the COVID-19 pandemic [5, 38]. Furthermore, the combination of protective practices in the household and online learning might worsen feelings of loneliness; a study of college students in the US showed a dramatic increase (71%) in stress and anxiety due to the COVID-19 pandemic [39]. Though these are different populations (school-aged vs. college age students), the study was conducted shortly after the move to online learning and reflects similarities in the challenges students were facing. Given that physical distancing was crucial to slow the spread of viral diseases, further research is needed to understand the possible synergies and feedback between online learning and psychological distress during a pandemic.

Thus, a pandemic paradox emerges: do we maximize protective practices and limit the spread of disease at the cost of higher risk of psychosocial distress, or do we risk overwhelming the health system and witness a spike in deaths, as we seek to protect children from potential psychological impact of a global pandemic? There is no simple answer, as both options have important negative impacts and implications. Extended quarantine can expose children to domestic violence [40–42], as well as exacerbate economic stress on the household, when childcare responsibilities prohibit a caregiver’s return to work. On the other hand, reopening schools not only exposes children to their own risk of infection, but the possibility of them infecting teachers, staff, and fellow students, which would only exacerbate negative psychosocial outcomes. Additionally, there are important disparities that will influence which children become infected: a recent study of pediatric COVID-19 cases found that 51% of the children infected were from low-income communities, while only 2% were from high-income communities [43]. Yonker et al. suggest that children from lower income settings pose a larger threat for introducing SARS-CoV-2 to their families as household size may be larger with multi-generational co-habitation and higher household density [43].

### Limitations

Data were not collected on household income; only loss of income due to COVID-19 was captured, thus limiting interpretation of some of these analyses. The survey instrument was designed in March 2020, when the use of masks was not widely recommended [44] and there were many uncertainties about the virus and its spread. As a result, only one question about mask usage was included in the knowledge section of the questionnaire. The survey was completed in mid-April when schools had already moved to remote learning and prevention campaigns were already in effect, which could explain the high rate at which knowledge questions were answered correctly. Response rate was low: of the 1178 students invited to participate (entire school), 280 successfully completed the questionnaire (23.7%). Though the school was targeted for inclusion in the study based on its socio-demographic representation of the state, the low enrollment may have introduced bias as there was an under representation of minorities enrolled in the study, when compared to school and state demographics.

## Conclusion

Many studies aim to predict how lockdown measures will flatten the pandemic curve, but few studies focus on the psychosocial impacts of these interventions on children and their families [5, 45–51]. As public health experts focus on reducing the spread of COVID-19 infections, it is imperative that they also focus on addressing the psychosocial needs of children. Additional research is needed to better understand and address the impacts of the pandemic and its societal responses on children, but doing so must be inclusive of vulnerable populations, including those whose households have lost income. Future research must include economic status and ensure diversity and inclusion of minorities in order to understand how the impacts affect vulnerable groups differently. Given that loss of household income was clearly an important risk factor for depressive, anxiety-related, and OCD-related symptoms, understanding a household’s baseline economic status becomes important in considering vulnerability and possible mitigating conditions. These findings have important implications for policy makers as they negotiate the continuation of funding for unemployment benefits and other safety nets for those economically disadvantaged, as losing these could increase the number of children at risk of negative psychosocial health outcomes. There should be a national, coordinated effort to continue collecting psychosocial data on children, as well as to design strategies and coping mechanisms for these children as societies across the country strive to balance the risks of COVID-19 infection and the risk of lockdown on psychosocial wellbeing. Previous disasters have shown that adverse psychological effects are not only present during the event, but also remain long after the incident [32]. Efforts to address mental health cannot wait until the pandemic is over, neither for school age population as the present study suggests, nor for adults, as other studies indicate [7, 9, 52–55]. At the very minimum, high risk groups must be identified across the US. These data contribute to evidence that lay bare the urgent need for primary and secondary school administrators, in collaboration with public health practitioners and medical professionals, to roll out targeted and group interventions as early as possible to respond to the emotional and psychosocial needs of children after a disaster occurs.

## Supplementary Information

**Additional file 1.** Timepoint 1 Questionnaire. PDF Questionnaire used to build the REDCap interface. The questions in this file were those asked to participants online after obtaining consent.

## Data Availability

Data may be requested from the PI of the project and corresponding author of this manuscript, Dr. Sarah L. McKune once it is released. The complete unidentified data set will be released after the completion of the longitudinal study (estimated date, August 2022).

## Abbreviations

aOR: Adjusted odds ratio
CI: Confidence interval
HIPPA: Health Insurance Portability and Accountability Act
KAP: Knowledge, attitudes, and practices
OCD: Obsessive compulsive disorder

## References

[1] Dong Y, Mo X, Hu Y, et al. Epidemiology of COVID-19 Among Children in China. Pediatrics. 2020;145(6). 10.1542/peds.2020-0702. 10.1542/peds.2020-0702 32179660

[2] Holmes* EA, O’Connor* RC, Perry VH, et al. Multidisciplinary research priorities for the COVID-19 pandemic. Lancet Pschyciatry. 2020;7(6):547–60. 10.1016/S2215-0366(20)30168-1 PMC7159850 32304649

[3] JJV B, Baicker K, Boggio PS, et al. Using social and behavioural science to support COVID-19 pandemic response. Nat Hum Behav. 2020;4. 10.1038/s41562-020-0884-z. 10.1038/s41562-020-0884-z 32355299

[4] Rolfsnes ES, Idsoe T. School-Based Intervention Programs for PTSD Symptoms: A Review and Meta-Analysis 2007;20(3):251–262. doi:10.1002/jts.20622. 10.1002/jts.20622 21425191

[5] Duan L, Shao X, Wang Y (2020). An investigation of mental health status of children and adolescents in China during the outbreak of COVID-19. J Affect Disord.

[6] Taquet M, Quoidbach J, Fried EI, Goodwin GM (2019). Mood homeostasis before and during the coronavirus disease 2019 (COVID-19) lockdown among students in the Netherlands. JAMA Psychiatry.

[7] Gassman-Pines A, Ananat EO, Fitz-Henley J (2020). COVID-19 crisis impacts on parent and child psychological well-being. Pediatrics..

[8] Xinyan X, Qi X, Yu Z, QI L, Jiajia Z, Ranran S. (2020). Mental health status among children in home confinement during the coronavirus disease 2019 outbreak in Hubei Province, China. JAMA Pediatr.

[9] Shi L, Lu ZA, Que JY (2020). Prevalence of and risk factors associated with mental health symptoms among the general population in China during the coronavirus disease 2019 pandemic. JAMA Netw Open.

[10] Werner PD. Implications of Attitude-Behavior Studies for Population Research and Action Author ( s ): Paul D . Werner Published by : Population Council Stable URL : https://www.jstor.org/stable/1966280 Implications of Attitude-Behavior Studies for Population Researc. 2020;8(11):294–299. 929668

[11] Ajzen I (2002). Perceived behavioral control, self-efficacy, locus of control, and the theory of planned behavior. J Appl Soc Psychol.

[12] Warwick D (1983). The KAP Survey: dictates of Mission versus demands of science.

[13] Zarocostas J (2020). How to fight an infodemic. Lancet.

[14] Chan EY, Cheng CK, Tam G, Huang Z, Lee P (2015). Knowledge, attitudes, and practices of Hong Kong population towards human a/H7N9 influenza pandemic preparedness, China, 2014 infectious disease epidemiology. BMC Public Health.

[15] Wang C, Pan R, Wan X (2020). A longitudinal study on the mental health of general population during the COVID-19 epidemic in China. Brain Behav Immun.

[16] Laurencin CT, McClinton A (2020). The COVID-19 pandemic: a call to action to identify and address racial and ethnic disparities. J Racial Ethn Heal Disparities.

[17] Reiss F (2013). Socioeconomic inequalities and mental health problems in children and adolescents: a systematic review. Soc Sci Med.

[18] McLoyd VC (1989). Socialization and development in a changing economy. Am Psychol.

[19] Alegria M, Vallas M, Pumariega A (2010). Racial and ethnic disparities in pediatric mental health. Child Adolesc Psychiatr Clin N Am.

[20] Kataoka SH, Zhang L, Wells KB (2002). Unmet need for mental health care among U.S. children: variation by ethnicity and insurance status. Am J Psychiatry.

[21] Jaycox L, Stein B, Kataoka SH (2002). Violence exposure, posttraumatic stress disorder, and depressive symptoms among recent immigrant schoolchildren. J Am Acad Child Adolesc Psychiatry.

[22] Cummings JR, Druss BG (2011). Racial/ethnic differences in mental health service use among adolescents with major depression. J Am Acad Child Adolesc Psychiatry.

[23] Slopen N, Fitzmaurice G, Williams DR, Gilman S (2010). Poverty, food insecurity, and the behavior for childhood internalizing and externalizing disorders. J Am Acad Child Adolesc Psychiatry.

[24] Breslau J, Aguilar-Gaxiola S, Kendler KS, Su M, Williams D, Kessler RC (2006). Specifying race-ethnic differences in risk for psychiatric disorder in a USA national sample. Psychol Med.

[25] Zhang AY, Snowden LR (1999). Ethnic characteristics of mental disorders in five U.S. communities. Cult Divers Ethn Minor Psychol.

[26] Mouzon DM (2013). Can family relationships explain the race paradox in mental health?. J Marriage Fam.

[27] Williams DR, González HM, Neighbors H (2007). Prevalence and distribution of major depressive disorder in African Americans, Caribbean blacks, and non-Hispanic whites: results from the National Survey of American life. Arch Gen Psychiatry.

[28] Zhong BL, Luo W, Li HM (2020). Knowledge, attitudes, and practices towards COVID-19 among chinese residents during the rapid rise period of the COVID-19 outbreak: a quick online cross-sectional survey. Int J Biol Sci.

[29] Gerhold L. COVID-19 : Risk perception and Coping strategies. Results from a survey in Germany. Interdiscip Secur Res Gr. 2020:1–11. https://psyarxiv.com/xmpk4.

[30] Kronenberg ME, Hansel TC, Brennan AM, Osofsky HJ, Osofsky JD, Lawrason B (2010). Children of Katrina: lessons learned about postdisaster symptoms and recovery patterns. Child Dev.

[31] McDermott BM, Palmer LJ (2002). Postdisaster emotional distress, depression and event-related variables: finding across child and adolescent development stages. Aust N Z J Psychiatry.

[32] Goldmann E, Galea S (2014). Mental health consequences of disasters. Annu Rev Public Health.

[33] Kaczynski KJ, Claar RL, Logan DE (2009). Testing gender as a moderator of associations between psychosocial variables and functional disability in children and adolescents with chronic pain. J Pediatr Psychol.

[34] Nolen-Hoeksema S, Girgus JS (1994). The emergence of gender differences in depression during adolescence. Psychol Bull.

[35] Lewinsohn PM, Lewinsohn M, Gotlib IH, Seeley JR, Allen NB (1998). Gender differences in anxiety disorders and anxiety symptoms in adolescents. J Abnorm Psychol.

[36] Kaltiala-Heino R, Marttunen M, Rantanen P, Rimpela M. Early puberty is associated with mental health problems in middle adolescence. Soc Sci Med 2003;57(6):1055–1064. 10.1016/S0277-9536(02)00480-X. 10.1016/s0277-9536(02)00480-x 12878105

[37] Angold A, Costello EJ, Worthman CM (1998). Puberty and depression: the roles of age, pubertal status and pubertal timing. Psychol Med.

[38] Kılınçel Ş, Kılınçel O, Muratdağı G, Aydın A, Usta MB. Factors affecting the anxiety levels of adolescents in home-quarantine during COVID-19 pandemic in Turkey. Asia-Pacific Psychiatry. 2020:1–6. doi:10.1111/appy.12406 10.1111/appy.12406 PMC7435562 32783389

[39] Son C, Hegde S, Smith A, Wang X, Sasangohar F (2020). Effects of COVID-19 on college students’ mental health in the United States: interview survey study. J Med Internet Res.

[40] Bradbury-Jones C, Isham L (2020). The pandemic paradox: the consequences of COVID-19 on domestic violence. J Clin Nurs.

[41] Campbell AM (2020). An increasing risk of family violence during the Covid-19 pandemic: strengthening community collaborations to save lives. Forensic Sci Int Reports.

[42] Humphreys KL, Myint MT, Zeanah CH. Increased risk for family violence during the COVID-19 pandemic. Pediatrics. 2020;146(1). 10.1542/peds.2020-0982. 10.1542/peds.2020-0982 32317306

[43] Yonker LM, Neilan AM, Bartsch Y, et al. Pediatric SARS-CoV-2: clinical presentation, infectivity, and immune responses. J Pediatr. 2020. 10.1016/j.jpeds.2020.08.037. 10.1016/j.jpeds.2020.08.037 PMC7438214 32827525

[44] CDC (2020). CDC ’ s recommendations for implementation of mitigation strategies for Florida , based on current situation with COVID-19 transmission and consideration of the state ’ s large older adult population.

[45] Ghosh R, Dubey MJ, Chatterjee S, Dubey S (2020). Impact of COVID −19 on children: special focus on the psychosocial aspect. Minerva Pediatr.

[46] Dubey S, Biswas P, Ghosh R (2020). Psychosocial impact of COVID-19. Diabetes Metab Syndr Clin Res Rev.

[47] Asbury K, Fox L, Deniz E, Code A, Toseeb U. How is COVID-19 affecting the mental health of children with special educational needs and disabilities and their families? J Autism Dev Disord. 2020. 10.1007/s10803-020-04577-2. 10.1007/s10803-020-04577-2 PMC7393330 32737668

[48] Oosterhoff B, Ph D, Palmer CA, et al. Since January 2020 Elsevier has created a COVID-19 resource centre with free information in English and Mandarin on the novel coronavirus COVID- 19 . The COVID-19 resource centre is hosted on Elsevier Connect , the company ’ s public news and information. Ann Oncol 2020;290(January):19–21.

[49] Liang L, Ren H, Cao R (2020). Correspondence. Psychiatr Q.

[50] van Gorp M, Maurice-Stam H, Teunissen LC, et al. No increase in psychosocial stress of Dutch children with cancer and their caregivers during the first months of the COVID-19 pandemic. Pediatr Blood Cancer. 2020:1–5. 10.1002/pbc.28827. 10.1002/pbc.28827 PMC7744828 33251717

[51] Stavridou A, Stergiopoulou AA, Panagouli E (2020). Psychosocial consequences of COVID-19 in children, adolescents and young adults: a systematic review. Psychiatry Clin Neurosci.

[52] Ettman CK, Abdalla SM, Cohen GH, Sampson L, Vivier PM, Galea S. Prevalence of Depression Symptoms in U.S. Adults Before and During the COVID-19 Pandemic. JAMA Netw Open. 2020;In Press(9). doi:10.1001/jamanetworkopen.2020.19686 10.1001/jamanetworkopen.2020.19686 PMC7489837 32876685

[53] Shim RS, Compton MT (2020). The social determinants of mental health: psychiatrists’ roles in addressing discrimination and food insecurity. Focus (Madison).

[54] Islam MA, Barna SD, Raihan H, Khan MNA, Hossain MT (2020). Depression and anxiety among university students during the COVID-19 pandemic in Bangladesh: a web-based cross-sectional survey. Plos One.

[55] Math S, Nirmala M, Moirangthem S, Kumar N (2015). Disaster management: mental health perspective. Indian J Psychol Med.

