# Supplementary material for: Psychosocial health of school-aged children during the initial COVID-19 safer-at-home school mandates in Florida: a cross-sectional study
Source: BMC Public Health. 2021 Mar 29;21:603. doi: 10.1186/s12889-021-10540-2 (PMC8006116; doi:10.1186/s12889-021-10540-2)
Supplement: Supplementary file 1 — Additional file 1. Timepoint 1 Questionnaire. PDF Questionnaire used to build the REDCap interface. The questions in this file were those asked to participants online after obtaining consent. [file 12889_2021_10540_MOESM1_ESM.pdf]

Date: _____________
Patient Name____
Address____
____

Contact information for follow-up: Name________phone ____________ e-mail _____________

Child Age (years) _______

Date of Birth (MM/DD/YYYY)_______

Child's biological sex Male ____ Female ____

How many years of school did <u>you</u> complete? _____ years

What type of health insurance does your child have?
- Private _____
- Medicare _____
- Medicaid _____
- None/Self-pay _____

Does your child have any of the following chronic illnesses?
- Seasonal allergies ______
- Diabetes _____
- Hypertension ______
- Cancer ______ Specify site: _______
- Heart disease ______
- Liver Disease ______
- Kidney Disease ______ Is s/he on dialysis? Yes ____ No _____
- Immunosuppression ______ If immunosuppressed, why? ______________
- Other _____ Specify: ____

<u>[If child is under 14]</u>
Does your child live with an adult who smokes cigarettes? Yes_____ No_____

If yes, how many packs does this person smoke per day? _______

[If child is over 13]
To your knowledge, does your child smoke cigarettes? Yes_____ No_____

To your knowledge, does your child use electronic cigarettes/vaping products? Yes _____ No ______

[Back to all]
What medications does your child take regularly (both prescription and "over the counter" non-prescription)?

**Medication**	**Dose (mg, if known)**	**Number of times you take it each day**

Flu history (influenza)
Has your child ever received a flu vaccine? (y/n)
Did your child receive the flu vaccine in the Fall of 2019 or Winter/Spring of 2020? (y/n)
If yes, injection or nasal?
Was your child diagnosed with the flu in the Fall of 2019 or Winter/Spring of 2020? (y/n)
If yes, when? (mm/dd/yy)
If yes, were they hospitalized for the flu ? (y/n)

Travel
Has your child traveled more than 20 miles outside of the county Since January 1, 2020? Yes ____ No ____
If yes, where and when? ____________________________
Add location (where & when)__________

Has your child traveled outside of the United States since January 1, 2020? Yes ____ No _____
If yes, where? ____________________________
Add location (where & when)_____

Has your child traveled to China since November 1, 2019? Yes ____ No _____
If yes, where did your child travel in China? ______________________________

Work
Is any adult in your child's home currently employed? Yes ____ No _____
If employed:
Full time____ Part time ______

Does anyone in your child's home work or volunteer in a hospital or other medical setting? Yes ____ No _____
If yes, where do they work/volunteer? _____________________________

If not in a medical setting, where does the adult work or volunteer?

Has a primary wage earner within your child's household lost their job or regular income source due to COVID-19? Yes ____ No _____
____

Home
Does your child live in an apartment complex or other multi-unit type of dwelling? Yes ____ No ____
If yes, provide details: ____________________________

SYMPTOMS
Please check off any symptoms that your child has had in the last 2 weeks, together with when they started and stopped

Symptom	Present Yes/ No? (for this illness period)	Start date	End date	Still present Yes/ No?
Fever*				
Cough				
Fatigue				
Shortness of breath				
Produces mucus when coughs				
Nasal congestion				
Sore throat				
Headache				
Joint or muscle aches				
Nausea				
Vomiting				
Diarrhea (3 or more loose stool in 24 hours)				
Abdominal Pain				
Loss of sense of taste				
Loss of smell				
Rash				
Other (specify)				

*If 'yes' to fever	Answer
Was it measured (yes/no)	
If measured, what method (oral/forehead/ear/armpit)	
Measurement value of the fever (F)	
Date of most recent fever	
Date that fever began for this illness period	

KNOWLEDGE, ATTITUDE, PRACTICES AROUND COVID-19

Please indicate if the following are true, false or if you don't know.

**Knowledge**	
K1. The main clinical symptoms of COVID-19 are fever, fatigue, dry cough, and muscle aches.	True, false, IDK
K2. Unlike the common cold, stuffy nose, runny nose, and sneezing are less common in persons infected with the COVID-19 virus.	True, false, IDK
K3. There currently is no effective cure for COVID-19, but early symptomatic and supportive treatment can help most patients recover from the infection.	True, false, IDK
K4. Antibiotics can be used to treat COVID-19	True, false, IDK
K5. Not all persons with COVID-19 will develop to severe cases. Those who are elderly and have chronic illnesses are more likely to be severe cases.	True, false, IDK
K6. People of all racial and ethnic groups can become infected with the COVID-19 virus.	True, false, IDK
K7. Most people who are infected with the COVID-19 virus recover from it.	True, false, IDK
K8. Handwashing can help reduce transmission of the COVID-19 virus.	True, false, IDK
K9. Persons with COVID-19 cannot pass the virus to others if they do not have symptoms.	True, false, IDK

K10. The COVID-19 virus spreads via respiratory droplets of infected individuals.	True, false, IDK
K11. Ordinary residents can wear general medical masks to prevent infection by the COVID-19 virus.	True, false, IDK
K12. It is not necessary for children and young adults to take measures to prevent infection by the COVID-19 virus.	True, false, IDK
K13. Isolation and treatment of people who are infected with the COVID-19 virus are effective ways to reduce the spread of the virus.	True, false, IDK
K14. People who have contact with someone infected with the COVID-19 virus should be immediately isolated in a proper place. In general, the observation period is 14 days.	True, false, IDK

Please indicate your level of agreement or disagreement with the following statements:

Attitudes

A1. I am worried about getting infected with the COVID-19 virus.	Agree, disagree, IDK
A2. I feel confident I can prevent myself and my family from becoming infected with the COVID-19 virus if it becomes more widespread in Florida.	Agree, disagree, IDK
A3. I know what actions to take to prevent myself and my family from becoming infected with the COVID-19 virus.	Agree, disagree, IDK
A4. I support a government-imposed mandatory quarantine for those who are infected with the COVID-19 virus.	Agree, disagree, IDK
A5. I support voluntary home quarantine for up to 2 weeks for people who have been in contact with someone who has COVID-19.	Agree, disagree, IDK
A6. I support postponing or canceling mass gatherings such as concerts, festivals, and sporting events.	Agree, disagree, IDK
A7. I support closure of K-12 schools if any student, staff member, or teacher is found to have COVID-19.	Agree, disagree, IDK
A8. If I were exposed to and could possibly be infected with the COVID-19 virus, I would be willing to quarantine myself at home for 2 weeks until I was sure I was not infected in order to prevent others from getting COVID-19 from me.	Agree, disagree, IDK

Please respond to each of the following questions by answering yes or no.

Practices

P1. In recent days, are you washing your hands with soap and water more often than normal?	Yes, no
P2. In recent days, are you using more disinfectants, such as hand sanitizers and cloth wipes?	Yes, no
P3. In recent days, are you avoiding shaking hands or other physical contact with others outside your home?	Yes, no
P4. In recent days, have you adhered to other social distancing guidelines, such as avoiding meetings of more than 10 people and keeping a distance of 6 feet apart?	Yes, no
P5. In recent days, have you bought larger amounts of staple foods (flour, sugar, pasta, rice, canned food) than normal?	Yes, no

CHILD STRESS and COPING MECHANISMS

If child is less than 8:
In consultation with your child, please have him/her answer the following questions:

In the past three weeks have you had the following feelings or thoughts? And if so, how often? (Never, A Little, Sometimes, A Lot, or Always; 1-5)

1. I feel hopeless and sad (about the virus)
2. I have trouble eating or sleeping
3. I find myself crying a lot
4. I feel worried or nervous (about the virus)
5. It is hard to stop my thoughts (about the virus)
6. I cannot stop worrying (about the virus)
7. I am very scared of getting dirty
8. I have to washing my hands, over and over to feel better
9. I have a stomachache/headache
I0. It’s hard for me to think a long time

Please ask and share your child’s thoughts on the following questions:

What do you feel when you think of the virus?
How can you help yourself feel better?
How would you help a friend if they were feeling scared about the virus?
How is school helping you with the virus?
What else could help you with your feelings about the virus?
Would you like to read about ways to feel better about the virus?

If child is 8-13:
Please allow your child to read and answer the following questions his/herself :

In the past three weeks have you had the following feelings or thoughts? And if so, how often? (Never, A Little, Sometimes, A Lot, or Always; 1-5)

1. I feel hopeless and sad (about the virus)
2. I have trouble eating or sleeping
3. I find myself crying a lot
4. I feel worried or nervous (about the virus)
5. It is hard to stop my thoughts (about the virus)
6. I cannot stop worrying (about the virus)
7. I am very scared of getting dirty
8. I have to washing my hands, over and over to feel better
9. I have a stomachache/headache
I0. It’s hard for me to think a long time

Please share your thoughts on the following questions:

What do you feel when you think of the virus?
How can you help yourself feel better?
How would you help a friend if they were feeling scared about the virus?
How is school helping you with the virus?

What else could help you with your feelings about the virus?
Would you like to read about ways to feel better about the virus?

If child is over 13:
Please allow your child to read and answer the following questions his/herself :

In the past three weeks have you had the following feelings or thoughts? (Never, Occasionally, Half the Time, Often, Always; 1-5))

1. Sadness, feeling down, low mood, feeling fatigued
2. Feelings of hopelessness, worthlessness, emptiness, or not being a good person
3. Decreased pleasure from things that used to be fun, feeling that life is not much fun
4. Feeling worried, nervous, panicky, tense, keyed-up
5. Not being able to stop worrying or controlling your worry
6. Being easily annoyed or irritable, feelings of dread like something awful might happen
7. Constant thoughts about avoiding germs
8. Fixation with washing your hands throughout the day
9. Sudden moments of fear or terror because you couldn't get rid of the germs
10. Felt a racing heart, shaky sweaty, or had trouble breathing

Please share your thoughts about the following questions:

If you feel stressed or scared about the coronavirus, what seems to help you feel better?
How do you help yourself feel calm when you hear about how the virus is spreading?
How do you help your friends that are feeling scared about the coronavirus?
What parts of school are helping you feel supported about your feelings about the coronavirus?
How does continuing to see your peers and teachers help you during this pandemic?
What types of handouts or readings would you be interested in about the coronavirus?

PARENTS STRESS and COPING MECHANISMS

In the past three weeks have you had the following feelings or thoughts? *(Never, Occasionally, Half the Time, Often, Always; 1-5)*

I worry about many different things
I am irritable and have a short temper
I have unexplained physical ailments (headaches, digestive problems, dizziness)
I am having a hard time: sleeping, eating, interacting with others positively

Problem focused coping
Please indicate whether you agree or disagree with the following statements as they relate to COVID-19 (1=strongly disagree, 5=strongly agree)

I listen to the experts and follow their advice.
I think carefully about what to do and stick to it.
I try not to do anything rash.
I focus on what to do next.
I focus on what I will do next.
I talk to others to learn more about the situation.
I know what to do and try to do everything with twice the effort.
I change things in my life to be able to cope better with it all.

I have repeatedly thought about it and try to understand it.
I have been thinking about what I usually do with other viral infections.
I talk to someone who knows about it.
I am doing something completely new that I would never have done in other circumstances.
I ask for advice from highly respected people and adhere to it.
I have seen something like this before.

Emotional coping

Please indicate whether you agree or disagree with the following statements as they relate to COVID-19 (1=strongly disagree, 5=strongly agree)

It will emerge over time; there is nothing more to do but wait.
I turn to my work or other activities to distract myself.
I imagine how the whole thing could end.
I imagine things that improve my mood.
I submit to my fate; sometimes you are just unlucky.
I tell myself things that make it easier for me.
I do things that are probably of no use, but I feel like at least I am doing something.
I wish I could change my worries and feelings.
I hope for a miracle.
I try to make myself feel better by eating, drinking, smoking or taking medication.
I take refuge in daydreams and imagine times when it was better than today.
I try to leave the whole thing behind and want to rest or go on vacation.
I refuse to believe what is happening.
